# Baiying qingmai formulation ameliorates thromboangiitis obliterans by inhibiting HMGB1/RAGE/NF-κB signaling pathways

**DOI:** 10.3389/fphar.2022.1018438

**Published:** 2022-10-11

**Authors:** Chongchong Zou, Li Liu, Chuanqi Huang, Song Hu

**Affiliations:** ^1^ Department of Pharmacy, Wuhan Hospital of Traditional Chinese and Western Medicine, Wuhan, China; ^2^ Department of Pharmacy, Liyuan Hospital, Tongji Medical College, Huazhong University of Science and Technology, Wuhan, China; ^3^ School of Pharmacy, Hubei University of Chinese Medicine, Wuhan, China

**Keywords:** thromboangiitis obliterans, Buerger’s disease, inflammation, traditional Chinese medicine, solanum lyratum

## Abstract

Baiying Qingmai Formulation (BF) is a classical clinical prescription used for decades to treat thromboangiitis obliterans (TAO). Although it effectively relieves pain and ischemic ulcers in patients with TAO, its anti-TAO mechanisms remain unclear. The chemical components of BF were analyzed using high-performance liquid chromatography and the potential targets of the compounds identified in BF were analyzed using molecular docking. Further, the signaling pathways and molecular mechanism of BF in treating TAO were studied using a rat model of TAO. Seven compounds (gallic acid, catechin, chlorogenic acid, caffeic acid, paeoniflorin, quercetin, and paeonol) were identified in BF, and molecular docking predicted their high affinities with HMGB1/RAGE/NF-κB proteins. In *in vivo* studies, BF not only inhibited the protein expression of HMGB1, RAGE, ICAM-1, and VCAM-1; mRNA levels of HMGB1 and RAGE; and the phosphorylation of NF-κB, ERK, Janus kinase (JNK) and p38 MAPK in the femoral artery, but also reduced the levels of inflammatory cytokines (IL-6, TNF-α, IL-1β, HMGB1) and stable metabolite (TXB2) of cytokine promoting thrombosis (TXA2) in the plasma. Moreover, BF stimulated the secretion of stable metabolite (6-keto-PGF1α) of cytokine inhibiting thrombosis (PGI2) in the plasma. BF inhibited the inflammatory response and thrombosis in the femoral artery, thus reducing the degree of vascular occlusion, which alleviated the symptoms in rats with TAO. Our findings suggest that BF ameliorates TAO by inhibiting the activation of the ERK, JNK, p38 MAPK and HMGB1/RAGE/NF-κB signaling pathways, thereby providing novel ideas for the treatment of TAO and essential information for the further development and utilization of BF as a promising drug to treat TAO.

## Introduction

Thromboangiitis obliterans (TAO) is a chronic inflammatory vascular disease that affects small- and medium-sized arteries and veins of the upper and lower extremities. Ulcers and gangrene of the limbs gradually occur as the disease progresses (Del Conde and Peña, 2014). Amputations are common because of extreme pain and necrosis of the limbs. The frequencies of major amputation were 11% at 5 years, 21% at 10 years, and 23% at 20 years, whereas minor amputations occurred at higher frequencies (Olin, 2018). Therefore, several patients are incapacitated, which is burdensome for themselves as well as society. The pathogenesis of TAO is unclear (Fazeli et al., 2021) and no effective treatments are currently known. Thus, the study on TAO and its etiology warrant more attention.

Clinical studies show the presence of inflammatory cell infiltration and thrombosis in the diseased vessels of patients with TAO (Halacheva et al., 2002; Olin and Shih, 2006; Li et al., 2013; Kobayashi et al., 2014). Inflammation and thrombosis are highly related (Zhou et al., 2020). The inflammatory response is considered to impair endothelial function and promote the development of TAO (Joras et al., 2006; Igari et al., 2017). Recent studies indicate that inflammatory cytokines such as interleukin (IL)-6, tumor necrosis factor-alpha (TNF-α), IL-1β, and high mobility group box-1 (HMGB1) were abnormally increased in the serum of patients with TAO (Dellalibera-Joviliano et al., 2012; De Caridi et al., 2016; Wu et al., 2018). HMGB1 elicits pro-inflammatory responses in various cells and is involved in the development of arteriosclerosis and systemic vasculitis, including Kawasaki syndrome, Churg Strauss syndrome, and Henoch-Schonlein purpura (Kang et al., 2014). It induces the release of inflammatory cytokines from neutrophils and macrophages (Al-Kuraishy et al., 2022), leads to IL-6 and TNF secretion by monocytes (Liu et al., 2018; Wei et al., 2018), and stimulates the production of TNF-α (Shapouri-Moghaddam et al., 2021), intercellular adhesion molecule-1 (ICAM-1), and vascular cell adhesion molecule-1 (VCAM-1) by endothelial cells (Fiuza et al., 2003). Therefore, HMGB1 may be a potential key target in the treatment of TAO (De Caridi et al., 2016; Mousazadeh et al., 2019).

The treatment of TAO is challenging owing to its unknown etiology. The drugs used to manage TAO mainly include cilostazol (Manolis et al., 2022), calcium antagonists, dextran, pentoxifylline, iloprost (Cacione et al., 2020), and bosentan (De Haro et al., 2012), which are associated with numerous side effects such as bleeding, a decline in fertility, bronchospasm, hypotension, and impaired liver function (Norona et al., 2020). Therefore, safe and effective drugs that can be used for a long period are required to decrease the disability rate.

BF is a classical prescription used in a clinical setting that was developed according to the basic theory of Traditional Chinese Medicine. It has been used to successfully treat TAO for decades with no obvious toxic or side effects (Sun and Liu, 2006). Thus, several patients with TAO have been either spared from amputation every year or have been able to significantly delay amputation. Although BF is safe and effective, its pharmacological mechanism in treating TAO has not been adequately studied. In this study, we first identified the chemical components of BF using high-performance liquid chromatography (HPLC). Next, the potential targets of the compounds identified from BF were analyzed based on molecular docking. Lastly, the anti-TAO mechanism of BF was analyzed using a rat model of TAO.

## Materials and methods

### Materials and reagents

Rat IL-1β (EK301BHS), TNF-α (EK382HS), and IL-6 (EK306/3) enzyme-linked immunosorbent assay (ELISA) kits were purchased from Multisciences Biotech (Hangzhou, Zhejiang, China). Rat HMGB1 (E-EL-R0505c), thromboxane B2 (TXB2, E-EL-R0965c), 6-keto prostaglandin F1 alpha (6-keto-PGF1α, E-EL-0054c) ELISA kits were procured commercially (Elabscience Biotechnology, Wuhan, Hubei, China). Rabbit polyclonal antibodies against HMGB1 (#6893S), receptor for advanced glycation end products (RAGE, #6996S), p-NF-κB p65 (#3033S), NF-κB p65 (#8242S), Janus kinase (JNK; #9252s), p-JNK (#4668s), ERK (#9102S), p-ERK (#4376s), p38 MAPK (#8690S), p-p38 MAPK (#4511S), and glyceraldehyde 3-phosphate dehydrogenase (GAPDH; #5174S) were purchased from Cell Signaling Technology (Beverly, MA, United States). Rabbit monoclonal antibodies against VCAM-1 (#ab134047) and mouse monoclonal antibodies against ICAM-1 (#ab171123) were purchased from Abcam (Cambridge, MA, United States).

### Preparation of BF

The 12 ingredients of BF (Table 1) were purchased from Hubei Tianji Pharmaceutical Company Ltd. (Jingzhou, Hubei, China) and soaked in 10 times their weight of purified water at 25°C for 1 h. The decoction was boiled for 2 h, and the residue was boiled again for 1.5 h with 10 times its weight of purified water. The two decoctions were pooled and filtered through eight layers of medical gauze. The filtrate was allowed to stand for 12 h and the supernatant was concentrated to 250 ml by using a rotary evaporator.

**TABLE 1:** composition of baiying qingmai formulation.

Chinese name	English name	Source	Weight (g)	Mass ratio	Voucher specimen number
白英	Solani lyrati herba	*Solanum lyratum* Thunb	65.2	3	20201201
牡丹皮	Moutan cortex	*Paeonia suffruticosa* Andr	21.7	1	20201202
赤芍	Paeoniae radix rubra	*Paeonia lactiflora* Pall	21.7	1	20201203
薏苡仁	Coicis semen	*Coix lacryma-jobi* L. var. *mayuen* (Roman.) Stapf	65.2	3	20201204
半枝蓮	Scutellariae barbatae herba	*Scutellaria barbata* D. Don	43.5	2	20201205
蛇莓	Duchesneae indicae herba	*Duchesnea indica* (Andr.) Focke	32.6	1.5	20201206
白花蛇舌草	Hedyotidis herba	*Hedyotis diffusa* Willd	43.5	2	20201207
紫草	Arnebiae radix	*Arnebia euchroma* (Royle) Johnst	65.2	3	20201208
徐長卿	Cynanchi paniculati radix et rhizoma	*Cynanchum paniculatum* (Bge.) Kitag	32.6	1.5	20201209
土茯苓	Smilacis glabrae rhizoma	*Smilax glabra* Roxb	65.2	3	20201210
川牛膝	Cyathulae radix	*Cyathula officinalis* Kuan	21.7	1	20201211
甘草	Glycyrrhizae radix et rhizoma	*Glycyrrhiza uralensis* Fisch	21.7	1	20201212

### Analysis of the chemical composition of BF using HPLC

HPLC is a common and effective analytical method for component identification. In this study, we identified the chemical components of BF by using HPLC. An LC-20AD system (Shimadzu Corp, Kyoto, Japan) equipped with an InertSustain C_18_ column (4.6 mm × 250 mm, 5 μm, GL Science Inc., Tokyo, Japan) at 30°C was used for analysis. The mobile phase was composed of A (methanol) and B (0.1% formic acid in water). The following gradient elution was used: 0–10 min, 5% A; 10–15 min, 10%–16% A; 15–20 min, 16%–30% A; 20–25 min, 30%–32% A; 25–40 min, 32% A; 40–45 min, 32%–72% A; 45–60 min, 72%–75% A; 60–65 min, 75%–5% A. UV absorption was determined at 280 nm, the injection volume was 10 μL, and the flow rate was 1.0 ml/min.

### Molecular docking

Molecular docking predicts the binding mode and the interactions between the acceptor-ligand complexes by studying their binding energy, action sites, and key residues. Hence, it is an important approach to predict and study the interaction pattern between small molecule ligands and protein receptors (Chen et al., 2020). In this study, molecular docking (AutoDock Vina; v1.1.2, National Biomedical Computation Resource, San Diego, CA, United States) was used to determine the binding activities and binding mode between the HMGB1/RAGE/NF-κB proteins and potential ligands. First, the 3D structures of HMGB1 (PDB: 6C1K), RAGE (PDB: 5D3F), and NF-κB (PDB: 2LWW) were downloaded from the RCSB protein databank (http://www.rcsb.org). The 2D structures of the components in BF were downloaded in the SDF format from the PubChem databank (https://pubchem.ncbi.nlm.nih.gov/) and transferred to the PDB format. Next, receptors and ligands were prepared by removing water molecules, adding hydrogen atoms, calculating the charges, and confirming the protonation state using Autodock Tools (v 1.5.6), and then converted into the readable pdbqt format. Subsequently, AutoGrid processing was performed to set the grid box. Lastly, AutoDock calculations were performed and PyMOL Molecular Visualization 2.3.2 (DeLano Scientific LLC, San Carlos, CA, United States) was used to observe and obtain all visual 3D geometry and docking results.

### Animals

Sterile-pathogen-free, adult male Wistar rats weighing 180–220 g were purchased from SPF Biotechnology Co., Ltd (Beijing, China) and housed at the Animal Experiment Center of the Hubei University of Traditional Chinese Medicine. All animal experiments were conducted strictly according to the Animal Ethics Committee at the Hubei University of Chinese Medicine (approval number: 202107003). All rats were housed at a temperature of 22 ± 2°C and relative humidity of 50%–60%, subjected to a 12-h/12-h light/dark cycle, and provided access to food and water *ad libitum*. Experiments were commenced 7 days after adaptive feeding.

### Grouping of rats and establishment of the TAO model

Sixty rats were randomized into the following six groups (*n* = 10): sham-operated group (Sham), TAO model group (TAO), BF low-dose group [BF-L, 3.85 g/kg, the clinic equivalent dosage according to the U.S. Food and Drug Administration (Nair and Jacob, 2016)], BF medium-dose group (BF-M, 7.71 g/kg), BF high-dose group (BF-H, 15.42 g/kg), and the Cilostazol group (68.6 mg/kg). Cilostazol (Otsuka Pharmaceutical Co., Ltd, Hangzhou, Zhejiang, China) was dissolved in 0.5% CMC-Na before gavage.

The rat model of TAO was established according to the method reported by Ashida et al (Ashida et al., 1980). Briefly, the right hind limbs of rats were depilated after anesthesia with an intraperitoneal injection of 150 mg/kg of 4% phenobarbital sodium (Lijiexun Pharmaceutical Co., Ltd, Fuzhou, Fujian, China). Next, a small incision was made at the groin. The femoral artery was exposed after removing the fat under the skin, and a clamp was used to block blood flow. Then, 0.15 ml of 1% sodium laurate (10 mg/ml in saline adjusted to pH 8.0, Sinopharm Chemical Reagent Co., Ltd. Shanghai, China) was injected into the distal end of the femoral artery. After 15 min, the clamp was removed and pressure was applied to stop the bleeding. Lastly, the skin was sutured and 0.1 ml of 400,000 units of penicillin sodium (Huabei Pharmaceutical Co., Ltd, Shijiazhuang, Hebei, China) was injected into the abdominal cavity to prevent infection. For rats in the Sham group, 0.15 ml saline instead of sodium laurate was injected into the femoral artery, and all other operations were the same as those conducted in rats in the TAO model group. After 12 h following the surgery, all rats were orally administered the test compounds once daily for 14 days. Rats in the Sham and TAO groups received a gavage of 0.5% CMC-Na solution. The pathological signs of the right hind limb were recorded daily and photographed every other day during the study.

### Histological examination

At the end of the experiment, the femoral arteries were isolated and soaked in 10% (v/v) neutral formalin at 4°C for 24 h. Tissues were embedded in paraffin, sliced into 4-μm-thick sections, and stained with hematoxylin and eosin (H&E). Infiltration of inflammatory cells and thrombosis were observed using an optical microscope equipped with a digital camera (BX53, OLYMPUS, Miyazaki, Japan).

### Analysis of cytokines in the plasma using ELISA

Enzyme-linked immunosorbent assay (ELISA) is one of the most specific and straightforward assays for detecting biomolecules in research and clinics. With advances in analytical methods, ELISA assay has been constantly optimized to improve its sensitivity, and different types of ELISA are now available to detect various biomarkers (Tabatabaei and Ahmed, 2022). At the end of the experiment, blood from the abdominal aorta was collected in an EDTA-containing anticoagulant tube. The plasma was centrifuged at 4°C for 15 min at 5,000 × *g*. IL-6, TNF-α, IL-1β, HMGB1, TXB2, and 6-keto-PGF1α levels in the plasma were determined using the corresponding ELISA kits according to the manufacturers’ instructions.

### Western blotting

Western blotting is an important procedure for the immunodetection of proteins (Kurien and Scofield, 2015), we studied the mechanism of TAO by assaying the expressions of many signalling pathway proteins and proinflammatory proteins. Proteins from the femoral artery were extracted using a RIPA kit (Merck KgAA, Darmstadt, Germany), separated using sodium dodecyl sulfate–polyacrylamide gel electrophoresis, and transferred to polyvinylidene fluoride membranes (0.45 μm, MILLIPORE, Boston, MA, United States). Next, the membranes were washed three times with Tris-buffered saline containing 0.1% Tween-20 (TBST) and incubated overnight at 4°C with the corresponding primary antibodies (dilution ratio of 1:2000). After washing three times with TBST, the membranes were incubated for 2 h at 37°C with horseradish peroxidase–conjugated secondary antibodies (dilution ratio of 1:4,000). The membranes were washed three times with TBST and incubated with enhanced chemiluminescence reagent (MILLIPORE, Boston, MA, United States). Lastly, protein bands were captured using X-ray films and the band intensities were measured with a Gel Imaging System (Bio-Rad, Hercules, CA, United States) using GAPDH as an internal reference.

### Analysis of mRNA expression using quantitative RT-PCR

The real-time polymerase chain reaction (RT-PCR) is the most sensitive method for the detection of low-abundance mRNA, often obtained from limited tissue samples. Quantitative RT-PCR has become the method of choice for gene expression analysis during the last few years (Wagner, 2013). In our study, total RNA from the femoral artery was extracted using Trizol reagent (Invitrogen, Carlsbad, CA, United States), and cDNA was synthesized using RNA as a template based on the instructions in the reverse-transcription kit (TaKaRa, Kyoto, Japan). The synthesized cDNA was amplified with specific primers (TaKaRa, Kyoto, Japan). The polymerase chain reaction (PCR) parameters were set as follows: denaturation at 95°C for 5 min; 40 cycles of 95°C for 15 s (denaturation); and 60°C for 31 s (annealing). Data were analyzed using the 2^−ΔΔCT^ method. Primer sequences are listed in Table 2.

**TABLE 2:** Sequences of quantitative real-time PCR primers.

Gene name	Sequence (5′→3′)
*HMGB1-F*	TCT​GTT​CTG​AGT​ACC​GCC​CA
*HMGB1-R*	GCG​GCC​TTC​TTT​TCA​TAG​GG
*RAGE-F*	CAG​TGT​GGC​TCG​AAT​CCT​CC
*RAGE-R*	ACT​TGA​CCT​CCT​TCC​CAA​GC
*GAPDH-F*	GCT​TCT​AGG​CGG​ACT​GTT​AC
*GAPDH-R*	CCA​TGC​CAA​TGT​TGT​CTC​TT

### Statistical analysis

Statistical analysis was performed by one-way analysis of variance (ANOVA), followed by the least significant difference test for calculating intergroup differences with SPSS 20.0 (SPSS Inc, Chicago, IL, United States). All data are presented as mean ± standard error of mean (SEM), and *p* < 0.05 was considered statistically significant.

## Results

### Identification of the components of BF

**FIGURE 1:**
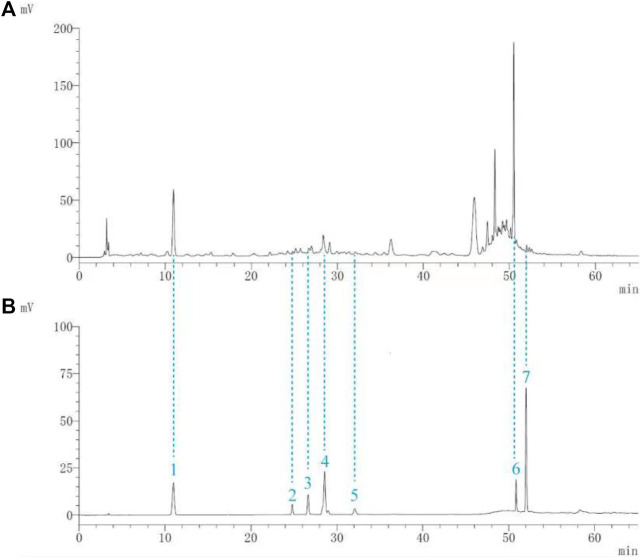
Identification of the components of Baiying Qingmai Formulation (BF) using HPLC. **(A)** Chromatogram of BF. **(B)** Chromatogram of the reference compounds: 1) gallic acid; 2) catechin; 3) chlorogenic acid; 4) caffeic acid; 5) paeoniflorin; 6) quercetin; 7) paeonol.

Seven components (gallic acid, catechin, chlorogenic acid, caffeic acid, paeoniflorin, quercetin, and paeonol) in BF were identified. The chromatographic peaks of the seven reference substances are shown in Figure 1B. The corresponding chromatographic peaks of the components of BF are identified in the chromatogram of BF (Figure 1A) and the contents are listed in Table 3.

**TABLE 3:** Concentrations (μg/ml) of the seven components in BF.

Components	BF-L	BF-M	BF-H
Gallic acid	90.75	181.50	363.00
Catechin	2.65	5.30	10.60
Chlorogenic acid	6.60	13.20	26.40
Caffeic acid	5.81	11.62	23.24
Paeoniflorin	21.96	43.92	87.84
Quercetin	8.15	16.30	32.60
Paeonol	1.67	3.34	6.68

### Molecular docking simulations

**FIGURE 2:**
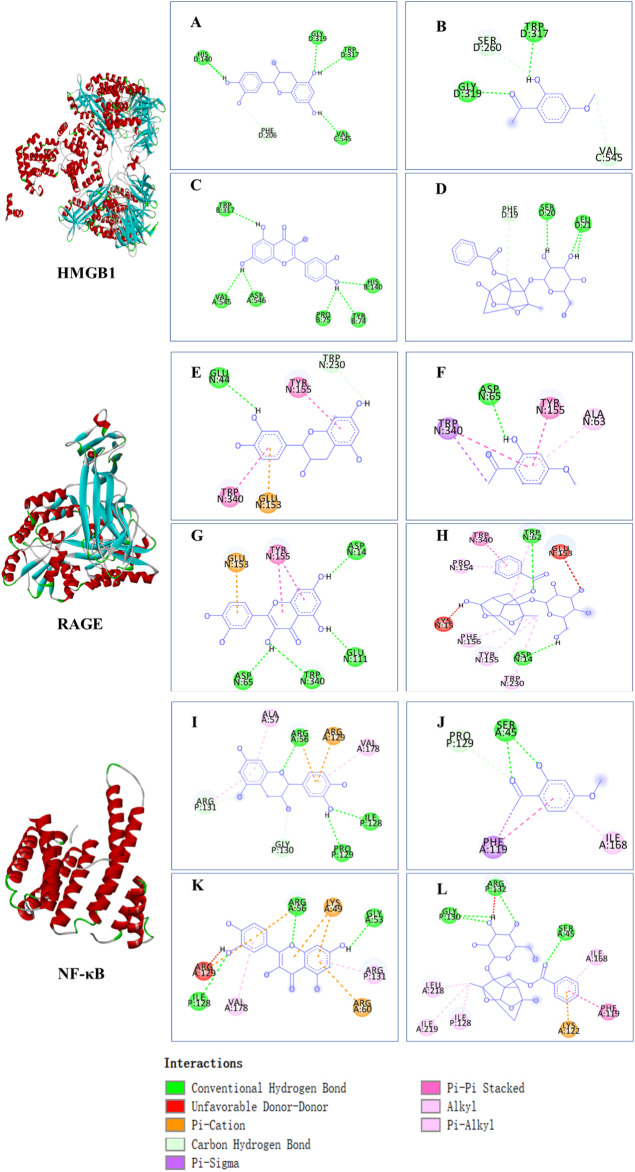
Molecular docking analysis of the putative binding. Binding modes between catechin, paeonol, quercetin, paeoniflorin, and HMGB1 (**A–D**), RAGE (**E–H**), and NF-κB (**I–L**), respectively. HIS, histidine; GLY, glycine; TRP, tryptophan; PHE, phenylalanine; VAL, valine; SER, serine; ASP, aspartic acid; PRO, proline; TYR, threonine; LEU, leucine; GLU, glutamic acid; ALA, alanine; LYS, lysine; ARG, arginine; ILE, isoleucine.

The affinities between the HMGB1/RAGE/NF-κB and the seven components of BF were lower than −5 kcal/mol (Table 4), which indicated high binding affinities and strong inhibitory activities toward HMGB1/RAGE/NF-κB proteins. Next, four components (catechin, paeoniflorin, quercetin, and paeonol) related to the effects of the drug were further stimulated with these proteins and their binding modes and active sites are shown in Figure 2.

**TABLE 4:** Molecular docking simulations between HMGB1/RAGE/NF-κB and the seven components of BF.

PubChem CID	Chemical structure	Ligand	Affinity (kcal/mol)		
			HMGB1	RAGE	NF-κB
370	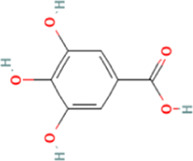	Gallic acid	−6.5	−6.0	−5.7
689043	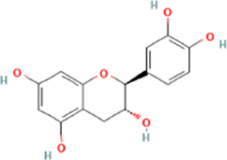	Catechin	−6.8	−6.3	−6.0
1794427	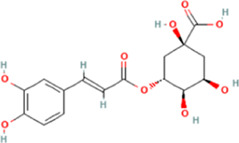	Chlorogenic acid	−8.7	−6.7	−6.8
9064	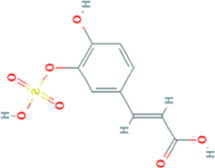	Caffeic acid	−9.2	−8.7	−7.7
442534	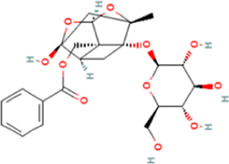	Paeoniflorin	−10.3	−9.7	−7.2
5280343	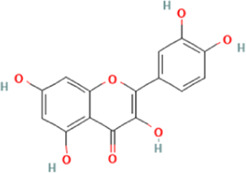	Quercetin	−9.3	−9.0	−7.4
11092	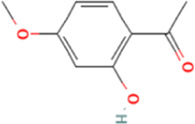	Paeonol	−6.4	−6.0	−5.8

### BF ameliorated symptoms of the right hind limb

**FIGURE 3:**
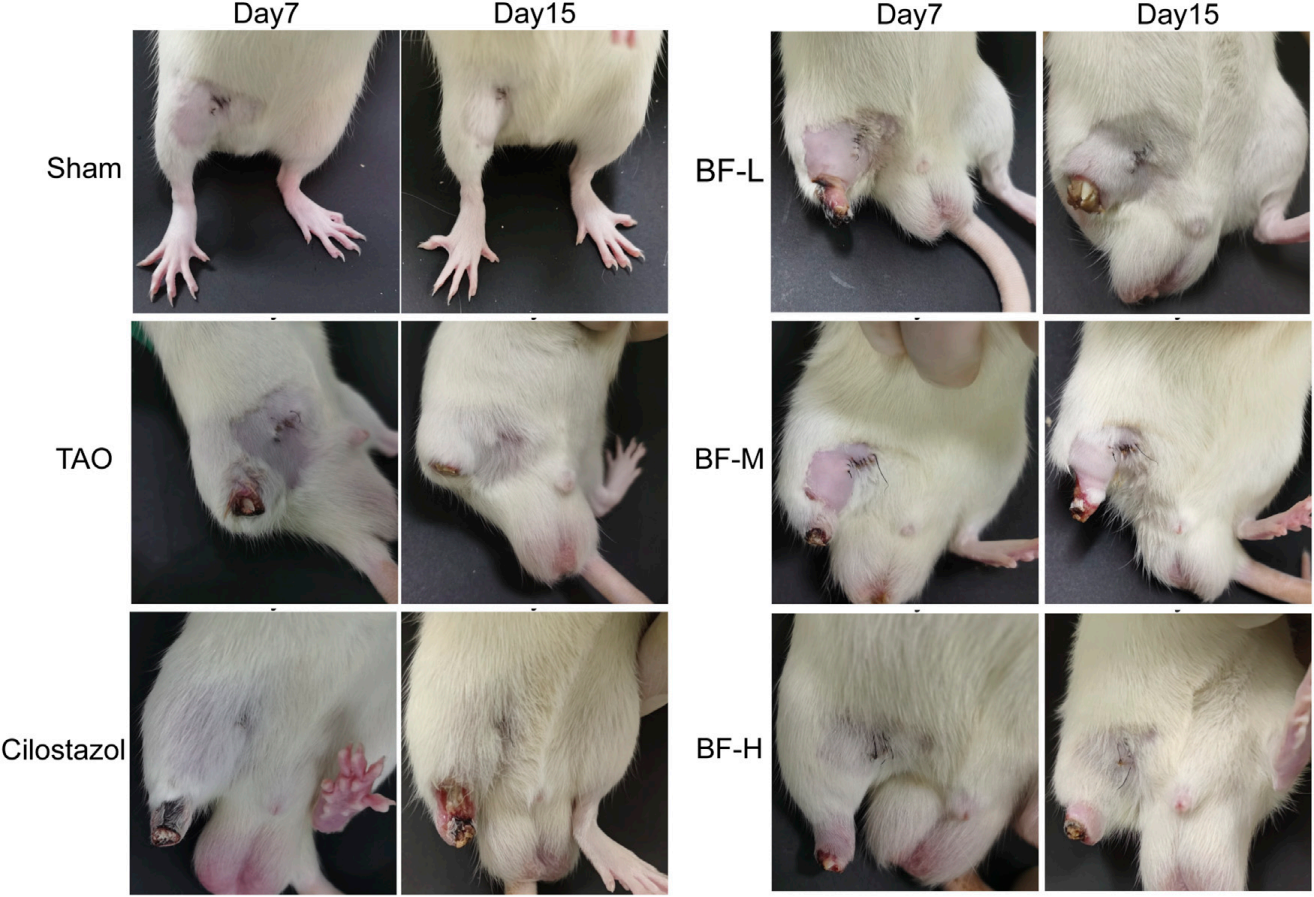
Appearance of the right hind limbs of rats. Sham: sham-operated group; TAO: TAO model group; Cilostazol: cilostazol group; BF-L: BF low-dose group; BF-M: BF medium-dose group; BF-H: BF high-dose group.

Rats in the Sham group were lively and exhibited normal activity throughout the experimental period. Rats in the other groups limped and showed ulceration and gradual gangrene formation on their right hind limbs (Figure 3), which was consistent with the symptoms exhibited by patients with TAO. On day 7, the thighs of rats in the TAO group fell off completely. After treatment with BF, a part of the thigh fell off in rats in the BF-L/M groups whereas only the paws fell off in those from the BF-H group. On day 15, the thighs of rats in the BF-L group fell off completely and ulcers occurred in the thighs of rats in the BF-M and Cilostazol groups. Treatment with BF-H ameliorated ischemic ulcers and prevented the limbs from falling off. Therefore, BF could inhibit the progression of TAO by ameliorating limb ischemia.

### BF ameliorated pathological symptoms of the femoral artery

**FIGURE 4:**
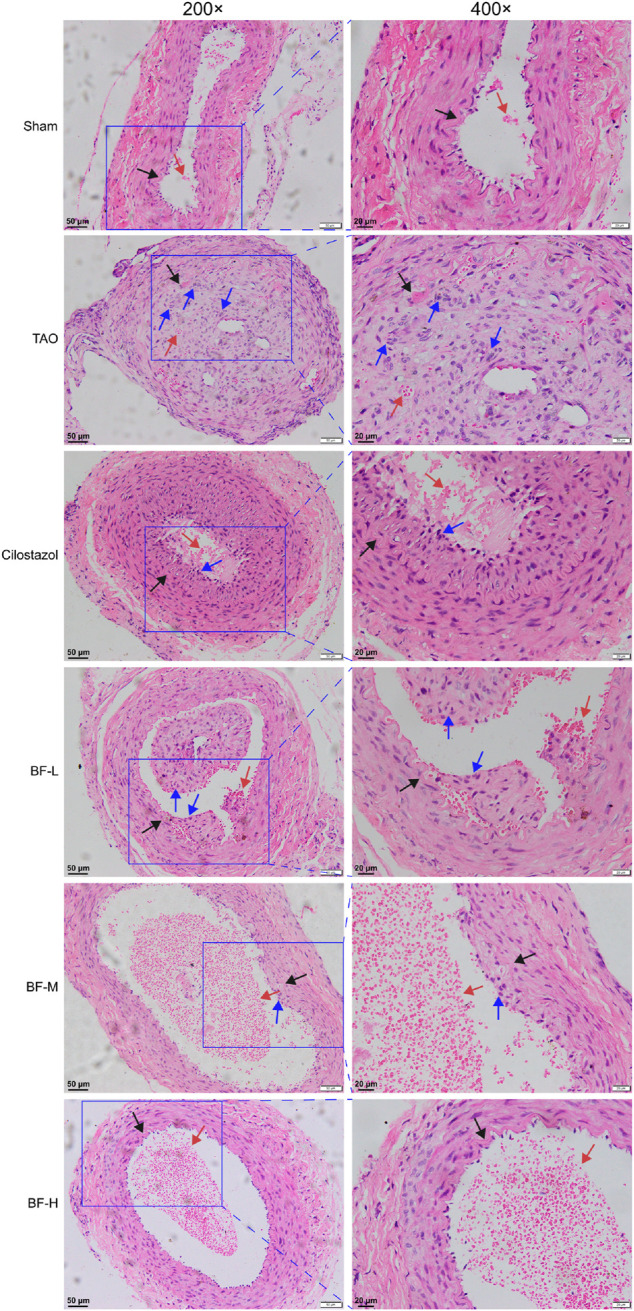
H&E staining of rat femoral artery. Black arrow: femoral artery intima; Red arrow: erythrocytes; Blue arrow: leukocytes. Sham: sham-operated group; TAO: TAO model group; Cilostazol: cilostazol group; BF-L: BF low-dose group; BF-M: BF medium-dose group; BF-H: BF high-dose group.

As shown in Figure 4, the intima of the femoral artery (black arrow) in the Sham group was intact. Although a few erythrocytes (red arrow) remained in the blood vessel, there was no thrombus, which was similar to that seen in unoperated rats in a previous study (Xu et al., 2009). The intima of the femoral artery of rats in the TAO group was severely damaged and the thrombus mainly consisted of inflammatory cells (blue arrow) clogging the vessel lumen. Although inflammatory cells adhered to the arterial walls of rats in the BF-L and Cilostazol groups, the intima of the femoral artery was intact and the degree of vascular occlusion was lower. Cellular inflammatory thrombus and adhesion of inflammatory cells to the vessel wall were significantly alleviated in rats in the BF-M and -H groups. Therefore, BF administration alleviated vascular occlusion in rats with TAO by inhibiting intravascular inflammatory reaction and cellular inflammatory thrombosis, thereby relieving limb ischemia.

### Effects of BF on plasma cytokines

**FIGURE 5:**
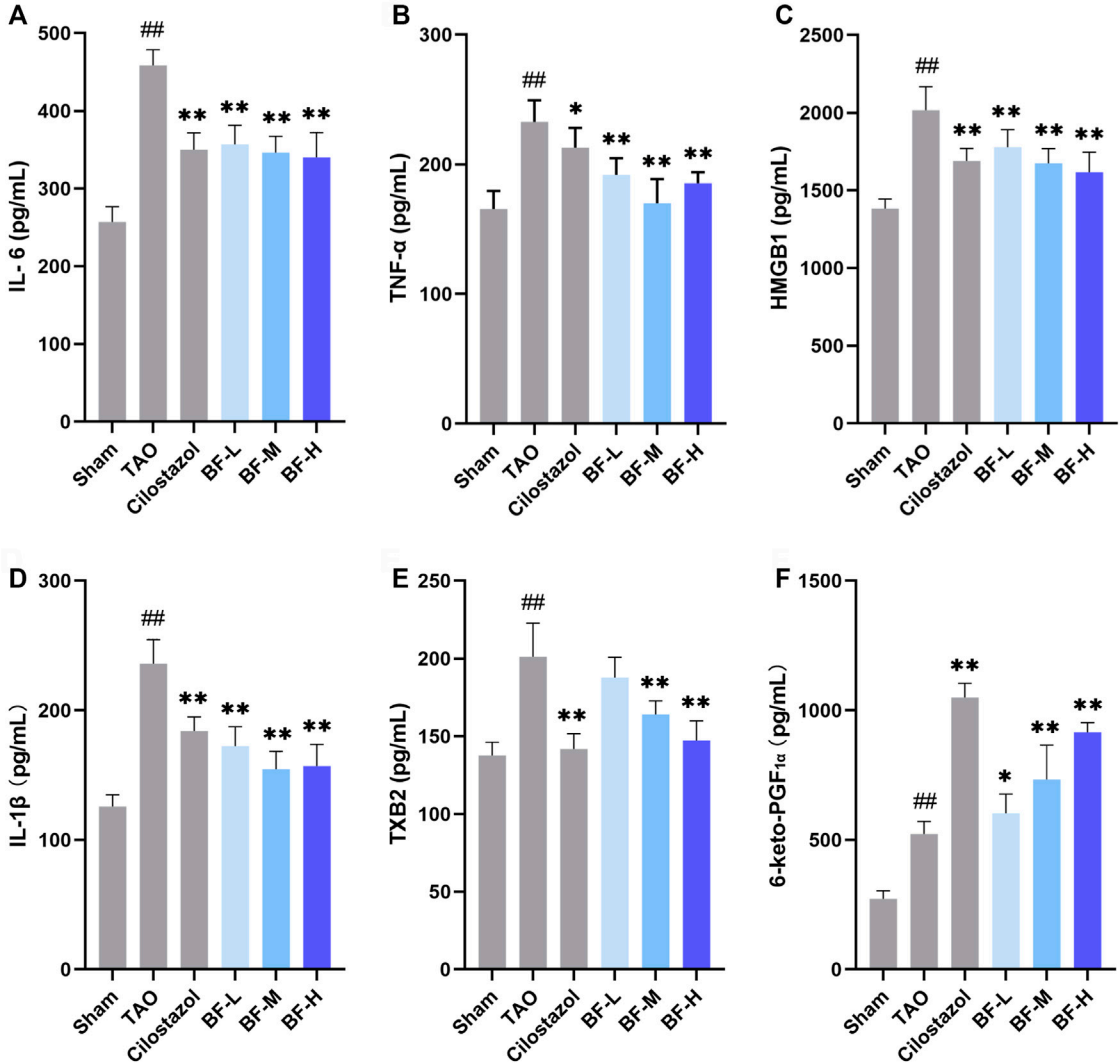
Effects of Baiying Qingmai Formulation (BF) on **(A)** IL-6, **(B)** TNF-α, **(C)** HMGB1, **(D)** IL-1β, **(E)** TXB2, **(F)** 6-keto-PGF1α levels in the plasma of rats with TAO. Data are presented as mean ± SEM (*n* = 6). #*p* < 0.05, ##*p* < 0.01 *versus* Sham, *p < 0.05, **p < 0.01 *versus* TAO. Sham: sham-operated group; TAO: TAO model group; Cilostazol: cilostazol group; BF-L: BF low-dose group; BF-M: BF medium-dose group; BF-H: BF high-dose group.

To determine the molecular mechanism of the anti-inflammatory and anti-thrombotic effects of BF, we measured the levels of plasma cytokines associated with inflammation and thrombosis. As shown in Figure 5, levels of IL-6, TNF-α, HMGB1, IL-1β, TXB2, and 6-keto-PGF1α were significantly increased in rats in the TAO group compared with those in the Sham group (*p* < 0.01). Treatment with low, medium, and high doses of BF remarkably decreased the levels of the pro-inflammatory cytokines IL-6, TNF-α, HMGB1, and IL-1β (*p* < 0.01; Figures 5A–D), indicating an anti-inflammatory effect. Moreover, TXB2 levels were significantly reduced in the BF-M/H groups (*p* < 0.01) (Figure 5E), whereas 6-keto-PGF1α levels were remarkably increased in a dose-dependent manner (*p* < 0.01) (Figure 5F). Therefore, BF alleviated TAO by regulating the expression of cytokines related to inflammation and thrombosis.

### Effects of BF on the expression of proteins in the femoral artery

**FIGURE 6:**
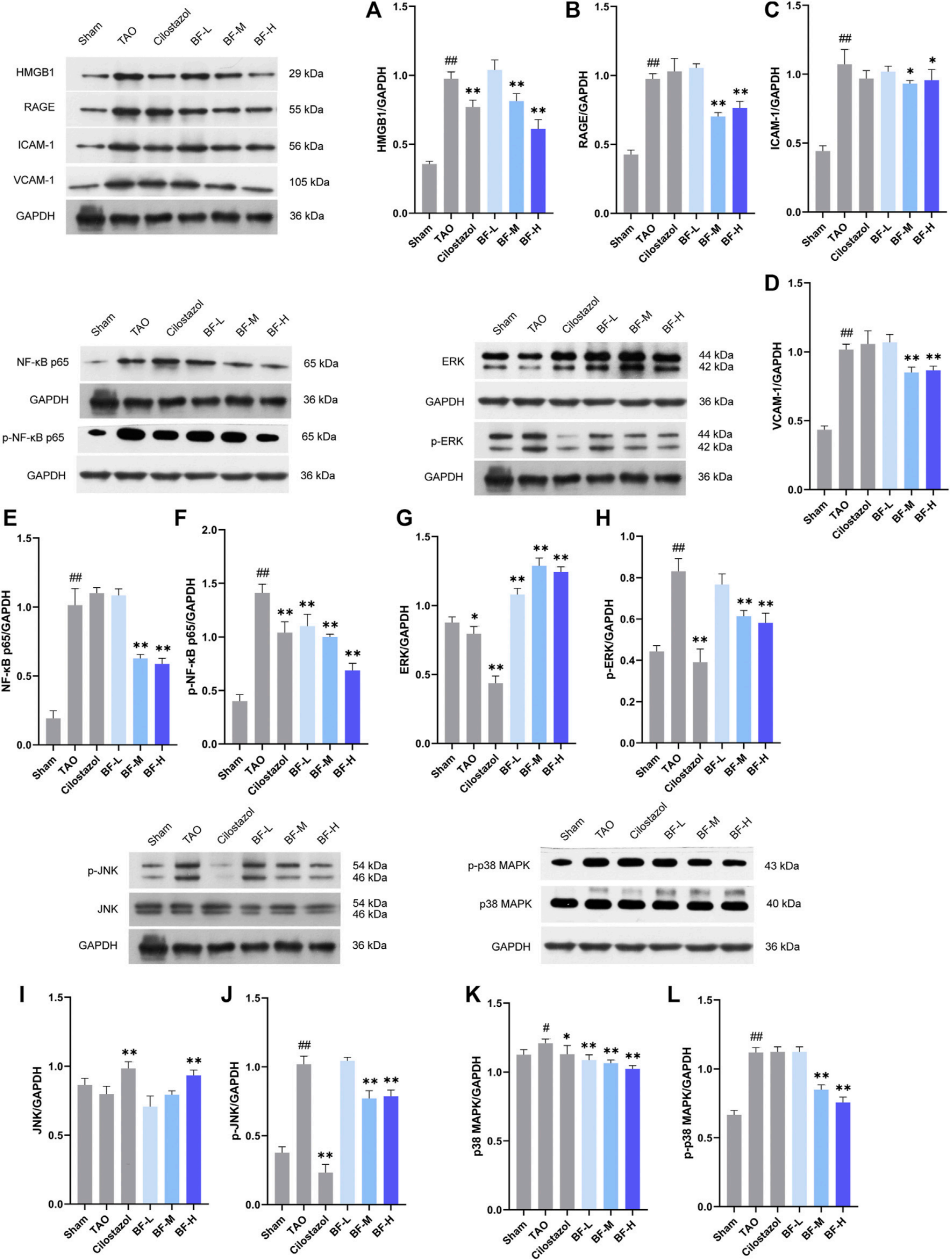
Effects of Baiying Qingmai formulation (BF) on proteins and signaling pathways of the inflammatory response. Levels of **(A)** HMGB1, **(B)** RAGE, **(C)** ICAM-1, **(D)** VCAM-1, **(E)** NF-κB p65, **(F)** phosphorylated NF-κB p65, **(G)** ERK, **(H)** phosphorylated ERK, **(I)** JNK, **(J)** phosphorylated JNK, **(K)** p38 MAPK, (**L**) phosphorylated p38 MAPK in the femoral artery. Data are presented as mean ± SEM (*n* = 3). #*p* < 0.05, ##*p* < 0.01 *versus* the Sham group, *p < 0.05, **p < 0.01 *versus* the TAO group. Sham: sham-operated group; TAO: TAO model group; Cilostazol: cilostazol group; BF-L: BF low-dose group; BF-M: BF medium-dose group; BF-H: BF high-dose group.

Several key proteins regulating the inflammatory response were identified using western blotting. HMGB1 (Figure 6A) and its receptor RAGE (Figure 6B) were significantly increased in the femoral artery of rats in the TAO group compared with those in the Sham group (*p* < 0.01) and were obviously reduced after treatment with BF-M/H (*p* < 0.01). Treatment with BF-M/H also suppressed the abnormal expression of ICAM-1 (Figure 6C), VCAM-1 (Figure 6D) and NF-κB p65 (Figure 6E) in rats with TAO. In the meantime, the phosphorylation level of NF-κB p65 was remarkablely reduced (Figure 6F). Along with the phosphorylation of ERK (Figure 6H) and JNK (Figure 6J), the levels of ERK (Figure 6G) and JNK (Figure 6I) were decreased in the TAO group, which suggested that a large number of ERK and JNK proteins are activated by phosphorylation, whereas treatment with BF-M/H led to notable inhibition of ERK and JNK signaling pathways in rats with TAO (*p* < 0.01). With respect to the p38 MAPK, BF-M/H inhibits its expression and activation at the same time (Figures 6K,L).

### Effects of BF on the mRNA levels of HMGB1 and RAGE in the femoral artery

**FIGURE 7:**
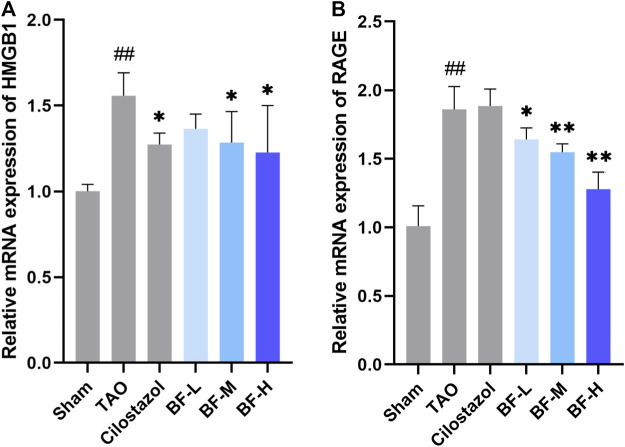
Impact of Baiying Qingmai Formulation (BF) on the mRNA levels of **(A)** HMGB1 and **(B)** RAGE in the femoral artery of rats with TAO. Data are presented as mean ± SEM (*n* = 3). #*p* < 0.05, ##*p* < 0.01 *versus* Sham, *p < 0.05, **p < 0.01 *versus* TAO. Sham: sham-operated group; TAO: TAO model group; Cilostazol: cilostazol group; BF-L: BF low-dose group; BF-M: BF medium-dose group; BF-H: BF high-dose group.

Next, we determined the mRNA levels of HMGB1 and RAGE using qPCR and found that their expression in the TAO group was remarkably higher than that in the Sham group (*p* < 0.01). Treatment with BF-M/H downregulated HMGB1 levels (*p* < 0.05) (Figure 7A) and remarkably decreased the mRNA expression of RAGE (*p* < 0.01) (Figure 7B).

## Discussion

Thromboangiitis obliterans is vasculitis characterized by intense cellular inflammatory thrombus (Piazza and Creager, 2010). Given the unclear pathogenesis of TAO, there are no specific drugs that can be used for comprehensive treatment. Thus, persistent treatment is essential to inhibit disease progression. An increasing number of studies on TAO have reported multiple risk factors such as the abnormal expression of pro-inflammatory cytokines (Shapouri-Moghaddam et al., 2019), smoking, infection by pathogens (Fazeli and Ravari, 2015), and an increased tendency toward blood coagulation to be related to the occurrence and exacerbation of TAO. Accordingly, inflammation, oxidative stress, immunity, and hemodynamic changes are considered to be their underlying mechanism (Li et al., 2020).

BF, prepared using 12 herbal medicines according to the basic theory of Chinese medicine, has the advantages of acting on multiple targets and exerting multiple effects due to the presence of complex compounds. Thus, it plays a significant role in relieving resting pain and ulcers and has been used clinically for decades to treat TAO without any reported adverse reactions (Sun and Liu, 2006). In this study, we identified seven components (gallic acid, catechin, chlorogenic acid, caffeic acid, paeoniflorin, quercetin, and paeonol) in BF (Figure 1) and used molecular docking to illustrate their high affinities with HMGB1/RAGE/NF-κB proteins (Table 4), which indicated their strong inhibitory activity on these proteins. Among the seven components, paeoniflorin inhibits NF-κB–mediated injury in human umbilical vein endothelial cells (Chen et al., 2018) and selectively inhibits platelet aggregation (Ngo et al., 2019), exhibiting favorable anti-inflammatory and anti-thrombotic effects. Quercetin is a phenolic flavonol with established antioxidant, anti-inflammatory, and immunostimulant properties (Imran et al., 2022). Owing to its anti-inflammatory effect, paeonol is used in the manufacture of drugs that are used to treat inflammation and pain (Zhang et al., 2019). Catechin can scavenge reactive oxygen species (ROS) and chelate metal ions. Its indirect antioxidant activities include the induction of antioxidant enzymes and inhibition of pro-oxidant enzymes, which may prevent and protect against diseases caused by oxidative stress (Bernatoniene and Kopustinskiene, 2018). So catechin, paeoniflorin, quercetin, and paeonol are consider to be the potential active ingredients according to these previous studies. Moreover, molecular docking manifested that the interactions between the four components and HMGB1/RAGE/NF-κB proteins were mainly by H bonding, a strong intermolecular interactive force. At the same time, pi-cations, alk1yl, and pi-alkyl interactions were involved (Figure 2), further enhancing the combination. As for gallic acid, caffeic acid, and chlorogenic acid, these phenolic acids get more attention on their anti-bacterial activity (Lima et al., 2016; Chen et al., 2022).

A large number of leucocytes constituting the thrombi of the femoral arteries in rats with TAO were found, indicating the successful establishment of the rat model of TAO. BF alleviated vascular occlusion by inhibiting intravascular inflammatory reaction and inflammatory thrombosis, especially in rats in the BF-M/H groups (Figure 4). Thus, ischemic symptoms in the right hind limbs were relieved after treatment with BF for 14 days (Figure 3), and TAO progression was inhibited as the dosage was increased. Endothelial cells, platelets, and leukocytes are the main components involved in the vascular inflammatory response and thrombosis. Several pro-inflammatory cytokines including IL-6, TNF-α, HMGB1, and IL-1β are abnormally high in the blood of patients with TAO (Fazeli et al., 2021; Shapouri-Moghaddam et al., 2021). An increase in IL-6 issues a warning signal during tissue damage or infectious lesions. Damage-associated molecular patterns (DAMPs) are released from damaged or dying cells and directly or indirectly promote inflammation (Tanaka et al., 2014). In this study, severe damage to the intima of blood vessels was found in rats with TAO. Meanwhile, IL-6 levels were remarkably increased in the blood of rats with TAO. BF intervention lowered IL-6 levels in a dose-dependent manner (Figure 5A) and maintained the integrity of the vascular intima (Figure 4). The DAMP HMGB1 is produced by various injured cells and is known to promote inflammation by binding to its receptor (RAGE and TLR2/4). During infection and upon exposure to inflammatory mediators, HMGB1 is actively secreted by monocytes, macrophages, natural killer cells, immature DCs, platelets, and the endothelium (Rapoport et al., 2020), leading to the activation of several signaling pathways and thereby modulating the inflammatory and immune responses. For instance, HMGB1 stimulates the secretion of inflammatory cytokines including TNF-α, IL-6, IL-1α, and IL-8 by monocytes and neutrophils (Ge et al., 2021). TNF-α is an important regulator of the inflammatory response that upregulates the expression of endothelial adhesion molecules, leading to the adhesion of monocytes to the endothelium (Evans et al., 2022). Therefore, HMGB1 maintains positive feedback with respect to the secretion of inflammatory cytokines. We found that BF-L/M/H intervention downregulated the increases in HMGB1, TNF-α, and IL-6 ( in the TAO group, suggesting that BF alleviated TAO by inhibiting HMGB1-mediated persiste Figures 5A–C) nt vascular inflammation. HMGB1 plays a role in the recruitment, activation, and adhesion of inflammatory cells to the vascular endothelium. It activates the adhesion and migration of monocytes and neutrophils (Rapoport et al., 2020). Stimulation by HMGB1 results in the increased expression of ICAM-1 and VCAM-1 in the endothelium, which elicits pro-inflammatory responses in endothelial cells and likely contributes to alterations in endothelial cell function in humans during inflammation (Fiuza et al., 2003). Several recent studies have reported the abnormal expression of HMGB1 in patients with TAO. For instance, high levels of HMGB1 were found in inflamed regions (Mousazadeh et al., 2019). RAGE, a receptor of HMGB1, is not highly expressed during normal physiological conditions; however, it is highly upregulated during chronic inflammation due to the accumulation of its ligands (Hudson and Lippman, 2018). BF-M/H intervention decreased both protein (Figures 6A,B) and mRNA (Figures 7A,B) levels of HMGB1 and RAGE in the femoral arteries of rats with TAO, thus decreasing the stimulation of endothelial cells by HMGB1 and thereby reducing ICAM-1 and VCAM-1 expression (Figures 6C,D). The overall outcome was inhibition of the recruitment, activation, and adhesion of inflammatory cells to the vascular endothelium, which eventually ameliorated vascular inflammation. Additionally, IL-1β can enhance the inflammatory response by inducing ICAM-1 expression on the surface of mesenchymal cells and VCAM-1 expression on the surface of endothelial cells (Dinarello, 2009). Treatment with BF-L/M/H effectively lowered IL-1β levels (Figure 5D).

By binding to its ligands, RAGE can activate multiple signaling pathways. The RAGE/NF-κB signaling pathway is involved in the pathogenesis of inflammatory diseases (Hudson and Lippman, 2018). In addition, HMGB1 activates pro-inflammatory effects in several cell types by binding to RAGE (Kang et al., 2013). Therefore, these pro-inflammatory effects may be mediated by NF-κB pathway. More specifically, NF-κB proteins are bound and inhibited by IκB proteins. Proinflammatory cytokines can activate an IKK complex which phosphorylates IκB proteins and then actives NF-κB proteins (Perkins, 2006). Next, they will be further activated by post-translational modifications (phosphorylation, acetylation, glycosylation) and translocate to the nucleus where it upregulates the expressions of TNF-α, IL-6, and IL-1β, which further activates the NF-κB signaling pathway and causes inflammatory cascade reaction (Huang et al., 2022). This study displayed that BF distinctly reduced both the expression and phosphorylation of NF-κB p65 (Figures 6E,F). Terefore, BF inhibited the activation of NF-κB to impede the entry of NF-κB into the nucleus, thereby alleviateing inflammatory response and ameliorating TAO.

In conjunction with the activation of NF-κB, MAPK activation induces the expression of multiple genes that together regulate the inflammatory response (Arthur and Ley, 2013). Moreover, inflammatory stimuli can also activate three MAPK pathways, which are mediated through ERK, JNK and p38 (Awasthi et al., 2021). For instance, the pro-inflammatory cytokines TNF-α, IL-6, IL-1β, and IL-17 are elevated in various states of chronic inflammation, where they interact with their respective receptors on the plasma membrane of several cell types to initiate the ERK signaling cascade (Lu and Malemud, 2019). ERK is predominantly present in two forms, ERK1 (p44) and ERK2 (p42). They are activated (i.e., phosphorylated) in the cytosol and subsequently translocated to the nucleus, where they activate transcription factors, thereby regulating diverse cellular events including proliferation, growth, differentiation, migration, survival, metabolism, and transcription. Similar to ERK, JNK proteins are highly responsive to an array of inflammatory cytokines. JNK regulates the cell cycle, adhesion, and migration through activation of the AP1 target genes in response to extracellular cytokines such as TNF-α (Hammouda et al., 2020). In the present study, BF-M/H effectively inhibited the expression of many pro-inflammatory cytokines, thereby inhibiting the phosphorylation of ERK and JNK, which blocked their cascade with NF-κB and inhibited the further progress of inflammatory response. With respect to p38 MAPK signaling pathway, it can be activated by inflammatory cytokines such as IL-1β and TNF-α. In addition, inhibitors of this pathway have been evinced to reduce inflammation. So it is considered to be the major signaling cassettes of the MAPK and pivotal regulator of inflammation (Awasthi et al., 2021). Although, whether MAPK pathways regulate other pathological processes remains unknown due to little research on the pathogenesis of TAO, it was confirmed that inflammation was critical for the occurrence or exacerbation of TAO. In this study, three MAPK pathways in TAO rats were activated by inflammatory stimuli and further regulated the inflammatory response, BF considerably reduced the levels of several pro-inflammatory cytokines, thereby inhibiting the activation of these MAPK pathways (Figures 6G–L), which resulted in the further downregulating of pro-inflammatory cytokines. Eventually, vascular inflammation was significantly alleviated.

The inflammatory response impairs endothelial function and accelerates the progression of TAO (Joras et al., 2006; Igari et al., 2017). More specifically, we believe that persistent vascular inflammatory response leads to the long-term activation of endothelial cells, which results in endothelial injury or dysfunction in patients with TAO. Subsequently, TXA2 is synthesized and discharged, thereby promoting vasoconstriction and platelet aggregation (Periayah et al., 2017) and contributing to thrombosis. Due to the instability of TXA2, biosynthesis is dependent on TXB2, a chemically stable and biologically inactive hydrolysis product of TXA2 (Patrono and Rocca, 2019). Treatment with BF-M/H reversed the increase in TXB2 in rats with TAO (Figure 5E). PGI2, secreted by endothelial cells, is a vasodilator and an inhibitor of platelet aggregation (Gu et al., 2021). We found that its hydrolysis product 6-keto-PGF1α was increased in rats in the TAO group, indicating that thrombosis triggers the antithrombotic mechanism. A further increase was observed after treatment with BF (Figure 5F). Therefore, BF was effective in inhibiting platelet aggregation.

## Conclusions

The mechanism by which BF alleviates ischemic ulcers and gangrene due to vascular inflammation and inflammatory thrombus has been elucidated in this study, which provides ideas and strategies for the treatment of TAO. Moreover, we examined the main active ingredients in BF prescription. Results from molecular docking indicate the strong affinities of these components with signaling pathways proteins. Collectively, our findings provide practical background information for the further development and utilization of BF to treat TAO. However, inaccurate identification of active compounds is the flaw of this study. Therefore, proteomics and metabolomics will be integrated into our consequent research.

## Data Availability

The datasets presented in this study can be found in online repositories. The names of the repository/repositories and accession number(s) can be found in the article/Supplementary Material.
